# Selective allylic hydroxylation of acyclic terpenoids by CYP154E1 from *Thermobifida fusca* YX

**DOI:** 10.3762/bjoc.10.137

**Published:** 2014-06-13

**Authors:** Anna M Bogazkaya, Clemens J von Bühler, Sebastian Kriening, Alexandrine Busch, Alexander Seifert, Jürgen Pleiss, Sabine Laschat, Vlada B Urlacher

**Affiliations:** 1 Institute of Technical Biochemistry, University of Stuttgart, Allmandring 31, 70569 Stuttgart, Germany; 2 Institute of Biochemistry, Heinrich-Heine University Düsseldorf, Universitätsstr. 1, 40225 Düsseldorf; 3 Institute of Organic Chemistry, University of Stuttgart, Pfaffenwaldring 55, 70569 Stuttgart, Germany

**Keywords:** allylic hydroxylation, cytochrome P450, natural products, protein engineering, regiochemistry, terpenoids

## Abstract

Allylic alcohols are valuable precursors in the synthesis of pharmaceutical intermediates, agrochemicals and natural products. Regioselective oxidation of parental alkenes is a challenging task for chemical catalysts and requires several steps including protection and deprotection. Many cytochrome P450 enzymes are known to catalyse selective allylic hydroxylation under mild conditions. Here, we describe CYP154E1 from *Thermobifida fusca* YX that enables this type of oxidation. Several acyclic terpenoids were tested as possible substrates for CYP154E1, and the regio- and chemoselectivity of their oxidation was investigated. Using a previously established bioinformatics approach we identified position 286 in the active site of CYP154E1 which is putatively involved in substrate binding and thereby might have an effect on enzyme selectivity. To tune regio- and chemoselectivity of the enzyme three mutants at position 286 were constructed and used for substrate oxidation. All formed products were analysed with GC–MS and identified using chemically synthesised authentic samples and known compounds as references. Best regioselectivity towards geraniol and nerol was observed with the wild type enzyme mainly leading to 8-hydroxy derivatives (8-hydroxygeraniol or 8-hydroxynerol) with high selectivity (100% and 96% respectively). Highest selectivities during the oxidation of geranylacetone and nerylacetone were observed with the following variants: V286F led mainly to 7-hydroxygeranylacetone (60% of the total product) and V286A produced predominantly 12-hydroxynerylacetone (75% of total product). Thus, CYP154E1 and its mutants expand the tool-box for allylic hydroxylation in synthetic chemistry.

## Introduction

Direct allylic hydroxylation yielding highly valuable allylic alcohols has been recognised for a while as one of the most oxyfunctionalizations [1]. Allylic alcohols can further be exploited for the synthesis of pharmaceutical intermediates, agrochemicals and natural products [2–4]. Various chemical catalysts including enzyme mimetica have been designed, characterised and applied for catalytic allylic hydroxylation reactions leading to synthetically relevant intermediates [5–11]. In addition to chemical catalysts a range of enzymes has been studied for selective allylic hydroxylation of alkenes [12–13]. Among biosynthetic routes selective allylic hydroxylation of monoterpene olefines to terpenoids in plants represents the most prominent example [14]. Heme-containing cytochrome P450 monooxygenases (P450 or CYP) are predominantly responsible for structural and functional diversity of terpenoids: allylic hydroxylation of parental monoterpenes leads to further diversification via sequential oxidation, reduction, isomerisation or conjugation reactions [14]. Furthermore, in some bacteria assimilating terpenes as carbon sources, the first oxidation step is a P450-mediated allylic hydroxylation or allylic rearrangement reaction [15]. In vitro investigations demonstrated that P450 enzymes can catalyse either the allylic hydroxylation of alkenes or the epoxidation of the corresponding C=C double bond or produce a mixture of the respective allylic alcohols and epoxides. Chemo- and regioselectivity of such reactions depend on the structure of the substrate and P450 used [16]. Different P450 enzymes produce different ratios of epoxidised and hydroxylated products [17–19]. The exact factors that govern the regiochemistry of P450 enzymes remain not completely understood [16]. In our previous studies we demonstrated the effects of the substrate stereochemistry on enzyme regio- and chemoselectivity [19]. The *E*-isomer geranylacetone was converted with a mutant of CYP102A1 from *Bacillus megaterium* (also referred to as P450 BM-3) with high activity and enantioselectivity to a single product 9,10-epoxygeranylacetone, while the oxidation of the *Z*-isomer nerylacetone yielded a mixture of several products, mainly epoxides but also allylic alcohols [19]. Later a CYP102A1 double mutant F87V/A328L was identified producing 80% allylic alcohols starting with geranylacetone [20]. These hydroxylated products are useful building blocks for the total syntheses of several natural compounds including smenochromene D [21], pseudopteranes, furanocembranes [22], indole alkaloids [23], and antitumor cembrane lactones crassin and isolobophytolide [24–25]. Obviously, there is a need for P450s with changed chemoselectivity.

Previously a systematic analysis of 31 P450 crystal structures and more than 6300 P450 sequences allowed us to derive rules on how to identify positions in the substrate binding cavity of P450s which is owing to its close proximity to the heme centre preferentially involved in substrate binding and thus in regioselectivity control [26]. Starting from two selectivity-determining positions, a minimal CYP102A1 library of only 24 variants was constructed and screened with four terpene substrates [20]. 11 variants demonstrated either a strong shift or improved regio- or chemoselectivity during oxidation of at least one substrate as compared to CYP102A1 wild type. This library was the starting point for engineering a highly selective CYP102A1 variant for terminal hydroxylation of (4*R*)-limonene at allylic C7 leading to perillyl alcohol. While the wild type did not hydroxylate (4*R*)-limonene at the C7 position, the triple mutant A264V/A238V/L437F converted (4*R*)-limonene to perillyl alcohol with a selectivity of 97% [27]. In a subsequent study, the effect of the two hotspot positions on regioselectivity towards cyclic and acyclic alkanes was investigated [28]. Among others, the double mutant F87V/A328F hydroxylated *n*-octane to 2-octanol with higher regioselectivity (92%) than the wild type (15%). To assess whether the concept of regioselectivity hotspots near the heme was transferable, residues that are equivalent to the two hotspot positions in CYP102A1 were mutated in CYP153A from *Marinobacter aquaeolei*. In the fatty acid ω-hydroxylase CYP153A, L354 corresponds to A328 in CYP102A1. While the wild type enzyme was highly ω-selective towards nonanoic acid with a ratio between the ω and the ω−1 product of 97:3, the variant L354I preferably hydroxylated nonanoic acid at the ω−1 position (24:76) [29].

Recently we reported on CYP154E1 from *Thermobifida fusca YX* that accepts a broad range of substrates including geraniol which is converted to 8-hydroxygeraniol [30]. In the present work several further acyclic terpenoids were screened as possible substrates for CYP154E1 and the regio- and chemoselectivity of their oxidation was investigated.

## Results

### Selection of the biocatalysts

**Figure 1:**
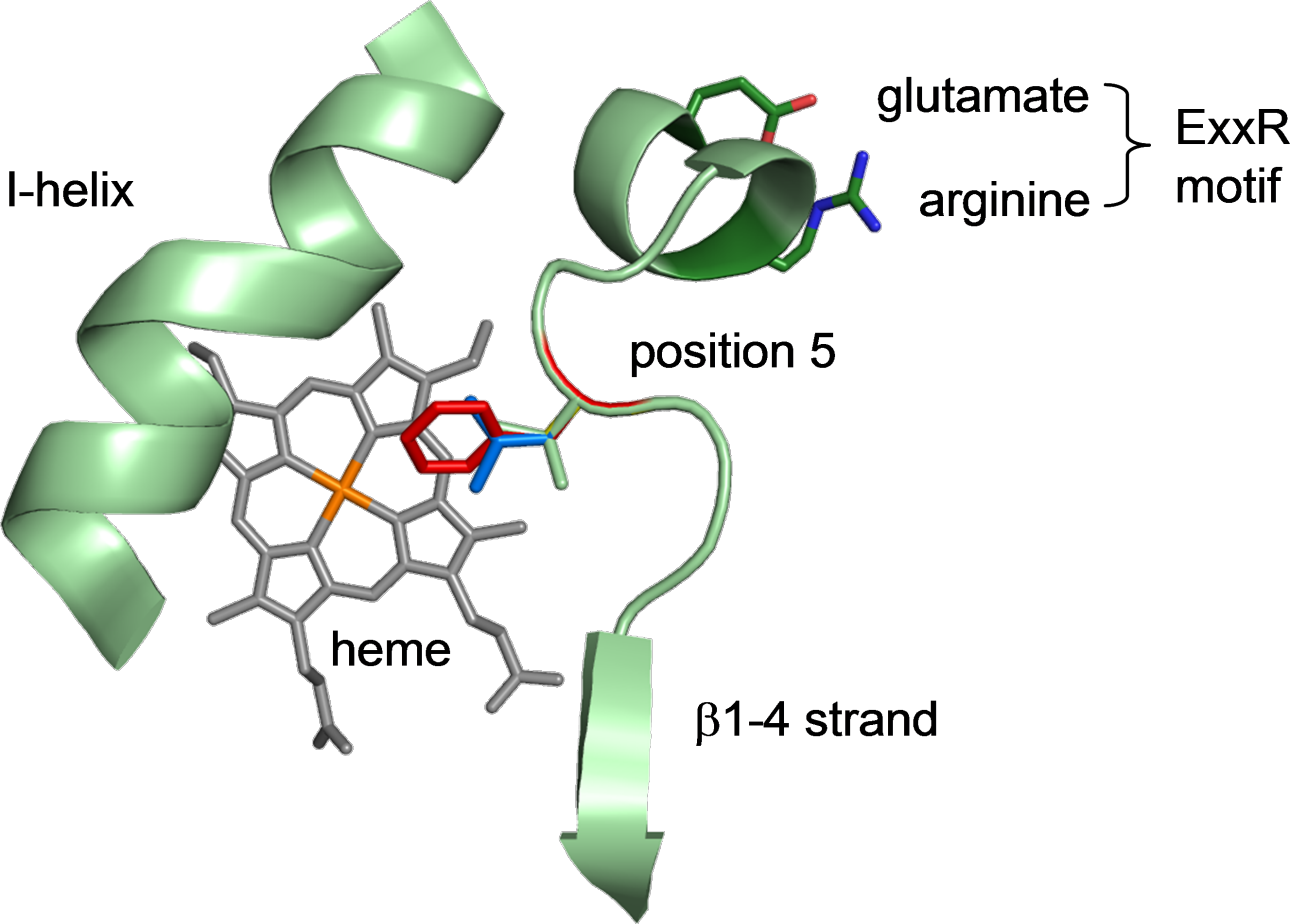
Immediate heme surroundings shown for the nearest relative of CYP154E1 with available crystal structure (CYP154A1, from *Streptomyces coelicolor*, PDB entry 1ODO). The residue in position 5 after the conserved ExxR motif (V286 in CYP154E1, green) reaches close to the heme centre (orange) in almost all P450s and was therefore substituted by phenylalanine (red), leucine (blue), and alanine (not visible). Based on the structure 1ODO point mutations were constructed using the software PyMol (DeLano Scientific LLC). From the rotamer library the candidates with the smallest sterical hindrance were selected.

According to the previously elaborated aforementioned sequence analysis, the residue at position 5 after the conserved ExxR motif is closest to the heme centre and therefore putatively interacting with any substrate in all P450s. In CYP154E1 V286 corresponds to position 5 after ExxR (Figure 1). The systematic analysis further revealed that residues at this position are predominantly hydrophobic. Hence, we substituted valine at position 286 by alanine, leucine and phenylalanine to change the orientation of substrates close to the heme centre.

**Scheme 1:**
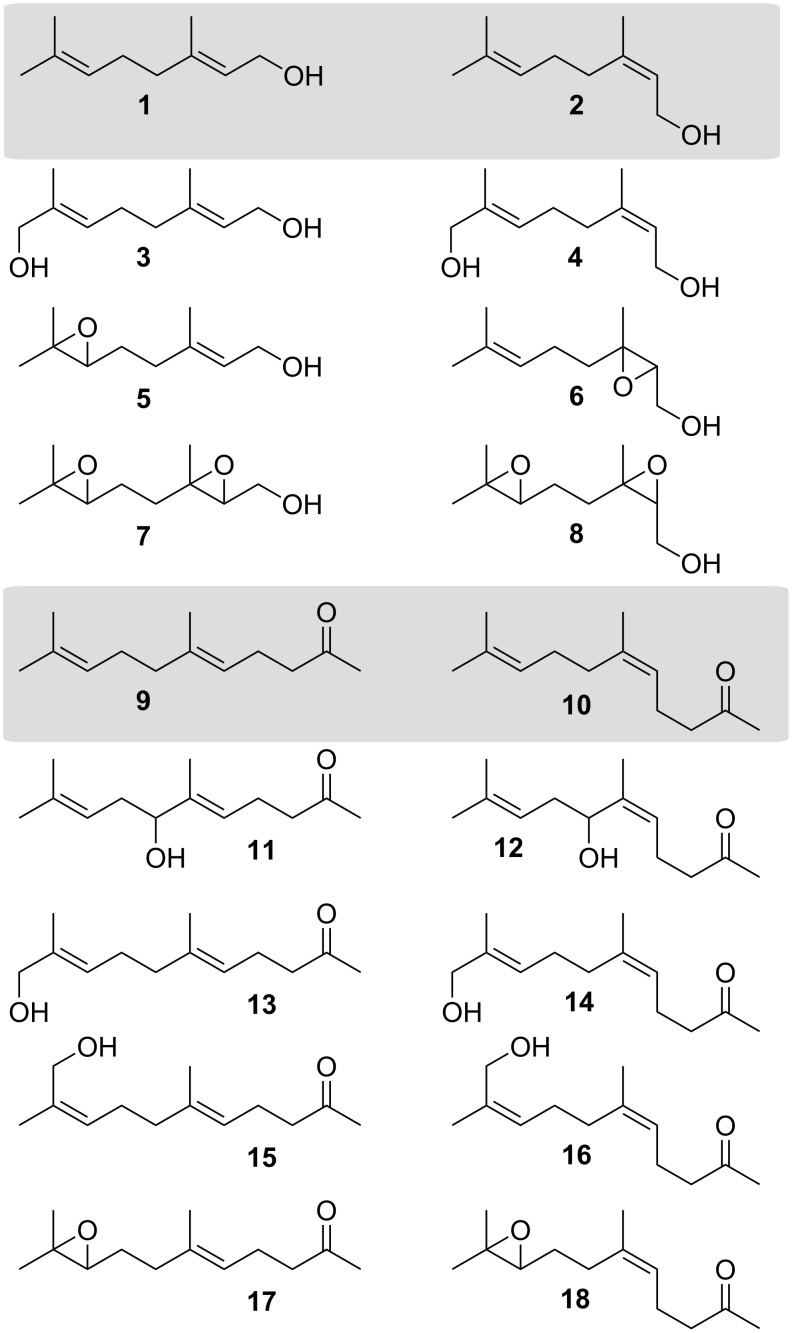
Terpene substrates (grey background) and their oxidised derivatives.

After soluble protein expression in *E. coli* and subsequent purification, catalytically active P450 systems were reconstituted by addition of putidaredoxin (Pdx) and putidaredoxin reductase (PdR) from *Pseudomonas putida* as well as the pyridine cofactor NADH. Reactions were performed in 500 µL reaction volume. In order to avoid the stoichiometric addition of NADH enzymatic regeneration of this expensive cofactor by glucose dehydrogenase was performed [31]. Reactions run for 4 h. Substrate conversion and product distribution were analysed by GC–MS (see Supporting Information File 1). Products were identified using chemically synthesised authentic samples and known compounds as references (Scheme 1).

### Chemical synthesis of the oxidation products

In order to identify the biocatalytic oxidation products synthetic routes to compounds **3**, **4**, **11**, **12**, **13** and **14** have been developed. The syntheses of compounds **5**, **7**, **8** [19] and **15–18** [20] have already been described before (see Supporting Information File 1).

**Scheme 2:**
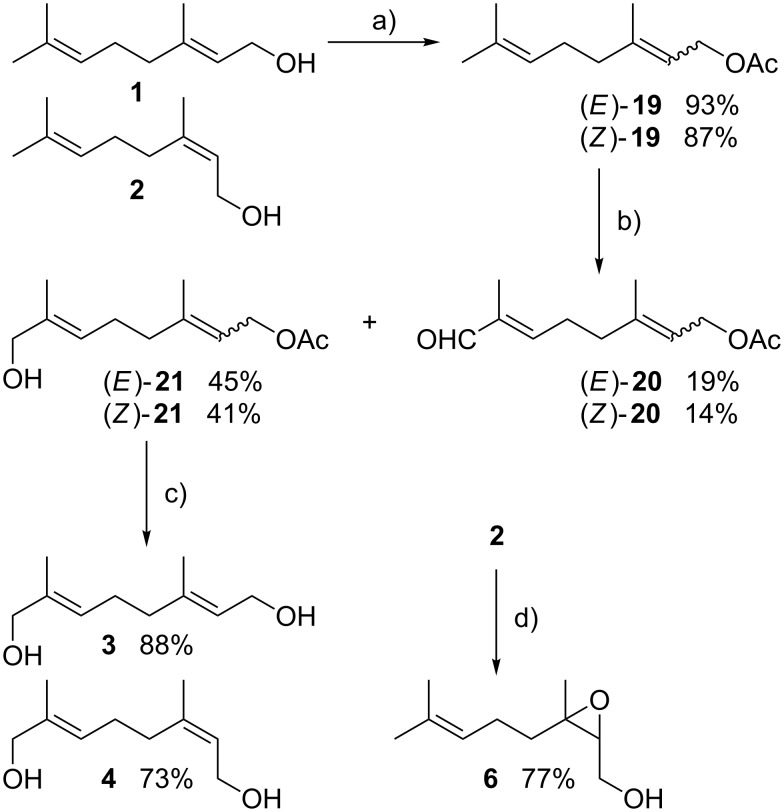
a) Ac_2_O, pyridine, room temperature, 24 h; b) SeO_2_, *t*-BuOOH, CH_2_Cl_2_, 0 °C, 5 h; c) K_2_CO_3_, MeOH, room temperature, 2.5 h; d) MPPA, NaOH, H_2_O, room temperature, 5 h (MPPA = magnesium monoperoxyphthalate).

There is no literature procedure for the synthesis of alcohol derivatives of geraniol (**1**) or nerol (**2**) by direct allylic oxidation. However, various conditions have been reported for geranyl acetate [32–34]. Following a modified procedure by Li [33], geranyl acetate ((*E*)-**19**), which was prepared from geraniol (**1**) in 93% yield [35] was treated under modified Sharpless conditions [36] with a catalytic amount of SeO_2_ in the presence of *t*-BuOOH in dichloromethane at 0 °C, to give enal (*E*)-**20** and allylic alcohol (*E*)-**21** in 19% and 45% yield, respectively, which were separated by column chromatography. The cleavage of the acetyl group was performed with potassium carbonate in methanol at room temperature [37]. Purification by chromatography led to the desired 8-hydroxygeraniol (**3**) in 88% yield (Scheme 2). A similar sequence was applied to nerol (**2**), which was converted into neryl acetate ((*Z*)-**19**) in 87%, followed by allylic oxidation [38], to provide enal (*Z*)-**20** and allylic alcohol (*Z*)-**21** in 14% and 41% yield, respectively. Saponification of 8-hydroxyneryl acetate ((*Z*)-**21**) under the above mentioned conditions gave 8-hydroxynerol (**4**) in 73% yield (Scheme 2). Following a procedure by Fringuelli et al. [39] nerol (**2**) was treated with magnesium monoperoxyphthalate (MPPA) in the presence of NaOH to give 2,3-epoxynerol (**6**) in 77% yield together with 20% of reisolated starting material **2** (see Supporting Information File 1).

**Scheme 3:**
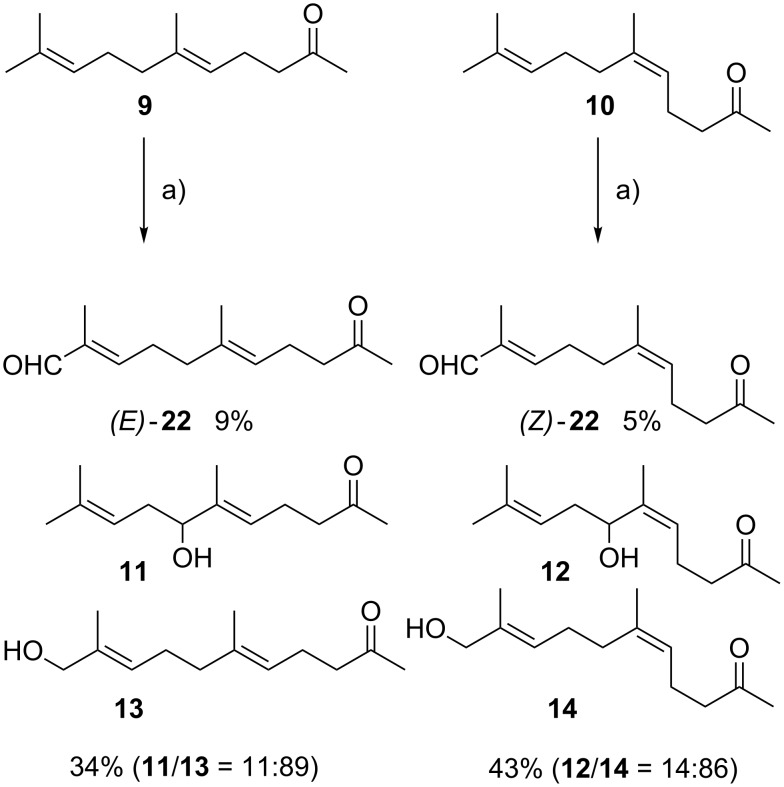
a) SeO_2_, *t*-BuOOH, CH_2_Cl_2_, 0 °C, 3.5 h.

According to a method by McMurry [24], modified Sharpless conditions [36] for the allylic oxidation of geranylacetone (**9**) were used. Purification of the crude product by chromatography yielded the enal (*E*)-**22** (9%) and a 11:89 mixture of the alcohols 7-hydroxygeranylacetone (**11**) and 11-hydroxygeranylacetone (**13**) in 34% yield and 11% of starting material **9** [24–25] (Scheme 3). The same procedure was employed for the oxidation of nerylacetone (**10**) providing after chromatography the enal (*Z*)-**22** (5% yield) and a 14:86 mixture of 7-hydroxynerylacetone (**12**) and 11-hydroxynerylacetone (**14**) in 43% yield and 4% **10** (see Supporting Information File 1).

### Oxidation of geraniol (**1**) and nerol (**2**)

The *E*- and *Z*-isomers geraniol (**1**) and nerol (**2**) were oxidised by CYP154E1 wild type and mutants. Geraniol (**1**) was oxidised by CYP154E1 (98% conversion) regio- and chemoselectively to a single product, which was identified as 8-hydroxygeraniol (**3**). Conversion of nerol (**2**) reached 77%, but was less selective than that of geraniol (**1**) resulting in the allylic alcohol 8-hydroxynerol (**4**, 96%) and 2,3-epoxynerol (**6**, 4%) as byproduct (Table 1).

**Table 1:** Comparison of geraniol (**1**) and nerol (**2**) oxidation catalysed by CYP154E1 wild type and three variants.

	Product distribution: Peak area^a^				Conversion	

	geraniol (**1**)		nerol (**2**)		**1**	**2**

CYP154E1	**3** (100%)		**4** (96%)	**6** (4%)	98**%**	77**%**
V286L	**5** (26%)	**7** (74%)	**8** (42%)	**6** (58%)	11**%**	4**%**
V286A	**5** (59%)	**7** (41%)	**8** (49%)	**6** (51%)	6**%**	3**%**
V286F	**5** (58%)	**7** (42%)	**8** (52%)	**6** (48%)	45**%**	22**%**

^a^Determined by GC–MS.

As expected, amino acid substitutions at position 286 changed both regio- and chemoselectivity of the wild type significantly. All three mutants V286L, V286A, and V286F showed strong preference for epoxides when starting with geraniol (**1**) and nerol (**2**), respectively. Mutant V286L was able to oxidise geraniol (**1**) to a mixture of 6,7-epoxygeraniol (**5**, 26%) and diepoxygeraniol **7** (74%) with 11% conversion, while nerol (**2**) was converted only up to 4% to get a mixture of 2,3-epoxynerol (**6**) and diepoxynerol (**8**) at a ratio of 58:42.

V286A and V286F catalysed the oxidation of geraniol (**1**) to a mixture of epoxides **5** and **7** as in the case of V286L. Two epoxide products appeared at a ratio of 59:41 (V286A), and only 6% substrate conversion was observed. The V286F mutant produced the same epoxides at a ratio of 58:42, however with a higher activity leading to substrate conversion of 45%.

Nerol (**2**) was oxidised by V286A and V286F again to epoxides **8** and **6**. In both cases conversions were rather low and reached 3 and 22%, respectively (Table 1).

### Oxidation of geranylacetone (**9**) and nerylacetone (**10**)

Geranylacetone (**9**) was oxidised by CYP154E1 wild type to four products with 98% conversion (Table 2). The main product was identified as 12-hydroxygeranylacetone (**15**, 42%). The other products were 11-hydroxygeranylacetone (**13**, 41%), 7-hydroxygeranylacetone (**11**, 8%) and the epoxide 9,10-epoxygeranylacetone (**17**, 9%). Similar to the conversion of geraniol (**1**)**,** the regio- and chemoselectivity of geranylacetone oxidation catalysed by the mutants was different compared to the wild type enzyme. The product analysis revealed a strong preference of all three mutants for the formation of C7-hydroxylated compound **11** as the main product.

**Table 2:** Comparison of geranylacetone (**9**) and nerylacetone (**10**) oxidation catalysed by CYP154E1 wild type and three variants.

	Product distribution: Peak area^a^		Conversion	

	geranylacetone (**9**)	nerylacetone (**10**)	**9**	**10**

CYP154E1	**11** (8%) **13** (41%) **15** (42%) **17** (9%)	**12** (12%) **14** (32%) **16** (56%)	98%	97%
V286L	**11** (44%) **13** (18%) **15** (35%) **17** (3%)	**12** (28%) **16** (72%)	20%	19%
V286A	**11** (46%) **13** (16%) **15** (15%) **17** (23%)	**12** (25%) **16** (75%)	25%	42%
V286F	**11** (60%) **13** (9%) **15** (31%)	**12** (24%) **14**(13%) **16** (63%)	75%	90%

^a^Determined by GC–MS.

Variant V286L produced 44% of 7-hydroxygeranylactone (**11**) as well as the compounds **13** (18%) and **15** (35%). Substrate epoxidation leading to the epoxide **17** was much slower compared to the wild type: this product accounted to less than 3% (substrate conversion was 20%). Variant V286A catalysed the hydroxylation of geranylacetone (**9**) to 7-hydroxygeranylacetone (**11**, 46%), 9,10-epoxygeranylacetone (**17**, 23%), 12-hydroxygeranylacetone (**15**, 15%) and 11-hydroxygeranylacetone (**13**, 16%) with a substrate conversion of 25%.

Variant V286F showed the strongest shift in regio- and chemoselectivity and converted geranylacetone (**9**) to a mixture of allylic alcohols without formation of epoxides. Substrate conversion was 75%, which is similar to that of the wild type. V286F led in this reaction to three allylic alcohols, again with a strong preference for C7 position (**11**, 60%) over C12 (**15**, 31%) and C11 (**13**, 9%).

The *Z*-isomer nerylacetone (**10**) was oxidised by CYP154E1 wild type and its mutants more selectively than the *E-*isomer geranylacetone (**9**) (Table 2). Three products were identified as a mixture of allylic alcohols with hydroxy groups incorporated at positions C7 (**12**, 12%), C11 (**14**, 32%) and C12 (**16**, 56%) with 97% conversion.

The product analysis of nerylacetone (**10**) oxidation catalysed with the mutants revealed a preference for the formation of hydroxylated products with 12-hydroxynerylacetone (**16**) as the main product. V286F oxidised nerylacetone (**10**) with high activity (90% conversion) leading to the same three allylic alcohols as the wild type enzyme, but with a shifted ratio towards 12-hydroxynerylacetone (**16**, 63%). Variants V286L and V286A showed even a stronger shift in regioselectivity and oxidised nerylacetone (**10**) almost exclusively to two allylic alcohols with a notable preference for 12-hydroxynerylacetone (**16**, 72% and 75%, respectively) (Table 2).

## Discussion

As mentioned in the introduction a large number of regioselective hydroxylation reactions occur in plants. Recently a geraniol 10-hydroxylase from the plant *Catharanthus roseus* [40] has been cloned and heterologously expressed in baculovirus-infected insect cells [41]. This cytochrome P450 monooxygenase belonging to the CYP76B family has been demonstrated to be involved into the biosynthesis of the alkaloid secologanin, and produced 8-hydroxynerol. It should be noted, that the enzyme used by the authors produced 8-hydroxynerol rather than 10-hydroxynerol according to their Figure 3 [41]. The use of this monooxygenase in biocatalysis is however not feasible because this enzyme is membrane bound, relies on a membrane associated cytochrome P450 reductase (CPR) and could only be produced in insect cells at low concentrations. Bacterial P450s have several advantages over their eukaryotic counterparts. They are soluble cytosolic enzymes interacting with soluble redox partner proteins. Thus, CYP154E1 from *T. fusca* is a valuable alternative for plant CYP76B because it is soluble and thermostable with a *T*_50_ of 63 °C. It interacts with also soluble redox partners and can be expressed in *E. coli* at a level of up to 50 mg/L. CYP154E1 is able to catalyse the regioselective hydroxylation of geraniol (**1**) to 8-hydroxygeraniol (**3**) and of nerol (**2**) mainly to 8-hydroxynerol (**4**). The chemical synthesis of these compounds requires 3 steps including protection/deprotection of the terminal hydroxy group.

Altering regio-, chemo- and stereoselectivity of P450-catalysed reactions remain a challenging task for protein engineering. In the recent years significant achievements have been made in this field. By using an iterative mutagenesis approach “combinatorial active-site saturation test (CAST)” Reetz and colleagues constructed several variants of CYP102A1 which demonstrate high regio- and stereoselectivity for testosterone and progesterone oxidation [42]. The parent F87A variant of CYP102A1 catalysed the hydroxylation of testosterone at both positions 2β and 15β. Simultaneous substitutions at three positions in the substrate binding pocket R47, T49, Y51 provided a variant yielding up to 94% of 2β-hydroxytestosterone. Combined mutations in the sites V78 and A82 favoured the 15β-position for hydroxylation and increased the enzyme selectivity towards 15β-hydroxylation finally up to 96% [42]. In another study Commandeur and colleagues tested a library of CYP102A1 variants for their ability to produce regio- and stereoselectively different diastereomers of 3-hydroxy-α-ionone. Several variants were identified with high selectivity for *trans*-3-hydroxy-α-ionone for both substrate enantiomers. They produced (3*R*,6*R*)-hydroxy-α-ionone and (3*S*,6*S*)-hydroxy-α-ionone. Other CYP102A1 mutants demonstrated opposite stereoselectivity and produced not only the trans-isomer (3*S*,6*S*)-hydroxy-α-ionone but also the *cis*-diastereomer (3*S*,6*R*)-hydroxy-α-ionone [43].

The identification of the position 286 in CYP154E1 that obviously influences the regioselectivity of the enzyme and subsequent replacing of valine at this position by three other hydrophobic amino acids of different size allowed for the production of epoxides. Although in this case both substrates, geraniol (**1**) and nerol (**2**) were epoxidised into two products respectively, variant V286F seems to be more promising as it produced 6,7-epoxygeraniol (**5**) at a product ratio of 58% with high activity.

Less selective was CYP154E1 during oxidation of geranylacetone (**9**) and nerylacetone (**10**). With geranylacetone as substrate three allylic alcohols and only 9% epoxide were formed. In the case of nerylacetone (**10**) the reaction catalysed by the wild type enzyme yielded three allylic alcohols. Though being less active, all three mutants were more regio- and chemoselective (except for V286A with geranylacetone (**9**)). Highest selectivities were observed with the following variants: V286F led mainly to 7-hydroxygeranylacetone (**11**, 60% of the total product) and V286A produced predominantly 12-hydroxynerylacetone (**16**, 75% of total product). Thus, to our knowledge for the first time P450s were engineered that catalyse the allylic hydroxylation of geranylacetone (**9**) mainly to 7-hydroxygeranylacetone (**11**) and nerylacetone (**10**) preferentially to 12-hydroxynerylacetone (**16**).

Interestingly, altered regioselectivity and high catalytic activity are not mutually exclusive properties. While for geraniol (**1**) and nerol (**2**) the mutants showed lower conversions than the wild type, for geranylacetone (**9**) and nerylacetone (**10**) the more selective mutant V286F had a catalytic activity similar to that of the wild type. A similar observation was made also during mutagenesis of CYP102A1 where high regioselectivity and high catalytic activity are not mutually exclusive properties. While for geranylacetone (**9**) the most selective mutant showed only 2% of the wild type activity, for limonene the most selective mutant had more than threefold wild type activity [20].

## Conclusion

Finally, in this study we investigated the application of CYP154E1 as regio- and chemoselective biocatalyst for the synthesis of allylic alcohols of acyclic terpenoids. Highest regioselectivity towards geraniol (**1**) and nerol (**2**) was observed with the wild type enzyme leading to mainly 8-hydroxy derivatives.

Moreover, by bioinformatics analysis position 286 was identified and a simple point mutation changed the geometry of the active site of this P450 enzyme to shift the product spectrum for the selective oxidation of geranylacetone (**9**) and nerylacetone (**10**) to yield only allylic alcohols.

## Supporting Information

File 1 Experimental and analytical data.
