# Supplementary material for: Selective allylic hydroxylation of acyclic terpenoids by CYP154E1 from Thermobifida fusca YX
Source: Beilstein J Org Chem. 2014 Jun 13;10:1347–53. doi: 10.3762/bjoc.10.137 (PMC4077532; doi:10.3762/bjoc.10.137)
Supplement: File 1 — Experimental and analytical data. [file Beilstein_J_Org_Chem-10-1347-s001.pdf]

Supporting Information

for

Selective allylic hydroxylation of acyclic terpenoids by CYP154E1 from *Thermobifida fusca* YX

Anna M. Bogazkaya[‡1], Clemens J. von Bühler[‡2], Sebastian Kriening[3], Alexandrine Busch[3], Alexander Seifert[1], Jürgen Pleiss[1], Sabine Laschat[3], Vlada B. Urlacher*[2]

Address: [1]Institute of Technical Biochemistry, University of Stuttgart, Allmandring 31, 70569 Stuttgart, Germany, [2]Institute of Biochemistry, Heinrich-Heine University Düsseldorf, Universitätsstr. 1, 40225 Düsseldorf, Germany and [3]Institute of Organic Chemistry, University of Stuttgart, Pfaffenwaldring 55, 70569 Stuttgart, Germany

*Corresponding author

Email: Vlada B. Urlacher - vlada.urlacher@uni-duesseldorf.de

[‡]Equal contributors

Experimental and analytical data

1. Chemicals

Monoterpenes and other chemicals were purchased from Fluka (Buchs, Switzerland) and Sigma (Deisenhofen, Germany). All chemicals were of analytical grade.

2. Biocatalyst preparation and biotransformations

2.1 Preparation of CYP154E1 monooxygenase, mutants and redox partners

Escherichia coli strain DH5α [F^-; *supE44*; *lacU169*; (Φ80d *lacZ M15*) *hsdR17*; *recA1*; endA1] was purchased from Clontech (Palo Alto, Calif., USA), *E. coli* strain BL21(DE3) {[B; F^- ; *omp*T *hsd*S_B (r_B- m_B^-); *dcm; galλ (DE3)*} and the pET-28a(+) vector were purchased from Novagen (Madison, Wis., USA). Genes of CYP154E1 from *Thermobifida fusca* YX, *camA* and *camB* (encoding for putidaredoxin reductase (PdR) and putidaredoxin (Pdx) genes from *Pseudomonas putida* ATCC17453, respectively) were amplified from pET-11a(+) from Mercian Corporation (Fujisawa, Japan), respectively, by PCR using primers designed to facilitate cloning into pET-28a(+) vector under control of the T7 phage promoter. The CYP154E1 gene was cloned as described earlier [1]. The three CYP154E1 mutants V286L, V286A, V286F were generated by using the "Quick change" site-directed mutagenesis method using the wild type gene as template. The following forward and reverse primers were used:

For V286L forward 5'- CTTCGAATCAGCGCTGGTCATGCTGCCGTTC-3',

reverse 5'-GAACGGCAGCATGACCAGCGCTGATTCGAAGCG-3';

For V286A: forward 5'- CTTCGAATCAGCGGCGGTCATGCTGCCGTTC-3',

reverse 5'- GAACGGCAGCATGACCGCCGCTGATTCGAAGC-3';

For V286F forward 5'- CTTCGAATCAGCGTTCGTCATGCTGCCGTTC-3',

reverse 5'- GAACGGCAGCATGACGAACGCTGATTCGAAGCG-3'.

The genes were amplified in 30 cycles of 2 min 95°C, followed by 2 min annealing at 57°C and extension for 4 min at 72°C in an Eppendorf thermal cycler. The amplified genes were subsequently cloned into the expression vector by *Nde*I/*EcoR*I restriction sites. Initial cloning has been performed in strain DH5α (Clontech, Heidelberg, Germany), which gives high transformation efficiency and good plasmid yield followed by heterologous expression in BL21(DE3) *E. coli* cells (Novagen , Madison, Wis., USA) which *N*-terminal His_6-tag to facilitate purification of the protein via nickel affinity chromatography. Attachment of the His_6 polypeptide had no effect on the activity of the gene product.

2.2. Protein expression and purification

Expression and purification by immobilised metal chelate affinity chromatography (IMAC) of CYP154E1 and its variants was performed as described earlier [1]. The redox partner proteins Pdx and PdR could be expressed in *E. coli* and purified by IMAC according to established protocols [2].The purity of the enzyme was estimated by SDS-PAGE, using 12.5% polyacrylamide gels. Enzyme samples were stored at -20°C until use.

2.3 Biotransformation and analysis

The 0.5 mL reactions were carried out in 2 mL plastic reaction tubes at 30°C. Reaction mixtures contained 0.2 mM substrate in 2% DMSO, 0.2 mM NAD^+, 10 μM P450 and 50 μM of each redox partner protein (Pdx and PdR) in 50 mM potassium phosphate buffer, pH 7.5. For NADH regeneration, the reaction mixture was

supplemented with 4 mM glucose-6-phosphate and 4 U/mL glucose-6-phosphate-dehydrogenase. After 4 h incubation time at 30°C, the reaction was stopped by addition of 20 µL of 37 % HCl. Reactions were supplemented with 50 µM (-)-carvone as internal standard and extracted with diethyl ether. Conversion was calculated based on peak areas.

Product analysis was performed via GC/MS on a Shimadzu GC/MS QP2010 (Kyoto, Japan) equipped with a FS-Supreme-5 column (0.25 mm x 30 m, 0.25 µm, CS-Chromatography Service, Langerwehe, Germany).

The column temperature was set to 120°C for 3 min and subsequently raised to 165°C at 5 K min^{-1}.The column temperature was then raised to 280°C with 30 K min^{-1} where it was held for 1 min. The temperatures of the injector and the interface were set to 285°C, respectively.

3. Chemical synthesis of terpenoids-derived oxidation products

3.1 Materials

All chemicals used in this work were purchased from Fluka (Buchs, Switzerland) or Sigma (Deisenhofen, Germany) and were of analytical grade or higher.

(*E*)-3,7-dimethyl-6,7-epoxy-2-octenol (6,7-epoxygeraniol) **5**, diepoxygeraniol **7**, diepoxynerol **8**, 9,10-epoxygeranylacetone **17** and 9,10-epoxynerylacetone **18** were prepared according to ref. [3], (5*E*,9*E*)-11-hydroxy-6,10-dimethylundeca-5,9-dien-2-one (11-hydroxygeranylacetone) **13**, (5*E*,9*Z*)-11-hydroxy-6,10-dimethylundeca-5,9-dien-2-one (12-hydroxygeranylacetone) **15**, (5*Z*,9*Z*)-11-hydroxy-6,10-dimethylundeca-5,9-dien-2-one (12-hydroxynerylacetone) **16** and (5*Z*,9*E*)-11-hydroxy-6,10-dimethylundeca-5,9-dien-2-one (11-hydroxynerylacetone) **14** were prepared as described in ref. [4].

3.2 Synthesis of reference compounds

General procedure for the allylic oxidation with selenium dioxide

Starting material (1.00 mmol) was added to a solution of selenium dioxide (44 mg, 0.40 mmol) and *t*-BuOOH (453 mg, 3.10 mmol) in dichloromethane (5 mL) at 0°C. After stirring under nitrogen at 0°C for a time *t* (*vide infra*), the mixture was diluted with ethyl acetate (15 mL), and washed successively with water (2 x 10 mL), saturated $NaHCO_3$ (10 mL), water (10 mL) and brine (10 mL). The organic layer was then dried over $MgSO_4$ and evaporated under reduced pressure. The crude product was purified by chromatography on silica gel (hexanes / ethyl acetate).

(*E*)-3,7-Dimethyl-octa-2,6-dienyl acetate (geranyl acetate) ((*E*)-19

Acetic anhydride (0.72 mL, 7.70 mmol) was added dropwise to an ice-cooled solution of geraniol (1.08 g, 7.00 mmol), dry pyridine (1.47 mL) in dichloromethane (10 mL). After warming to room temperature, the mixture was stirred for 24 h. Water (20 mL) and dichloromethane (20 mL) were then added and the aqueous layer was extracted with dichloromethane (20 mL). The combined organic layers were washed successively with saturated $NaHCO_3$ (10 mL) and brine (10 mL), dried and evaporated under reduced pressure to yield geranyl acetate (*E*)-**19** as a colorless oil (1.28 g, 6.5 mmol, 93%). ^{1}H NMR (500 MHz, $CDCl_3$): $\delta = 5.36$-5.33 (m, 1H, 8-H), 5.10-5.07 (m, 1H, 4-H), 4.59 (d, J = 7.0 Hz, 2H, 3-H), 2.12-2.08 (m, 2H, 7-H), 2.06-2.03 (m, 2H, 6-H), 2.05 (s, 3H, 1-H), 1.70 (s, 3H, 12-H), 1.68 (s, 3H, 10-H), 1.60 (s, 3H, 11-H) ppm. ^{13}C NMR (125 MHz, $CDCl_3$,): $\delta = 171.1$ (2-C), 142.3 (5-C), 131.9 (9-C), 123.8 (8-C), 118.3 (4-C), 61.4 (3-C), 39.5 (6-C), 26.3 (7-C), 25.7 (10-C), 21.1 (1-C), 17.7 (11-C), 16.5 (12-C) ppm. Spectroscopic data were in accordance with ref. [5].

(2*E*,6*E*)-8-Acetoxy-2,6-dimethylocta-2,6-dienal ((*E*)-20

Oxidation of geranyl acetate (396 mg, 2.00 mmol) with selenium dioxide according to the general procedure (t = 5 h) gave after purification by column chromatography (silica gel, eluent: hexanes / ethyl acetate 7 : 3) in a first fraction (R_f = 0.6) (*E*,*E*)-8-acetoxy-2,6-dimethyloct-2,6-dienal (*E*)-**20** as a colorless oil (79 mg, 0.38 mmol, 19%) and in a second fraction (R_f = 0.3) 10-hydroxygeranylacetate (*E*)-**21** as a colorless oil (191 mg, 0.90 mmol, 45%). ^{1}H NMR (300 MHz, $CDCl_3$): δ = 9.39 (s, 1H, CHO), 6.47-6.42 (m, 1H, 3-H), 5.41-5.36 (m, 1H, 7-H), 4.59 (d, J = 7.1 Hz, 2H, 8-H), 2.51-2.46 (m, 2H, 4-H), 2.26-2.21 (m, 2H, 5-H), 2.05 (s, 3H, 10-H), 1.75 (m, 6H, 11,12-H) ppm. ^{13}C NMR (125 MHz, $CDCl_3$): δ = 195.2 (1-C), 171.1 (9-C), 153.4 (3-C), 140.4 (C_q), 139.7 (C_q), 119.6 (7-C), 61.1 (8-C), 37.8 (5-C), 27.0 (4-C), 21.0 (10-C), 16.4 (11-C), 9.2 (12-C) ppm. Spectroscopic data were in accordance with ref. [6].

(2*E*,6*E*)-3,7-Dimethyl-8-hydroxyocta-2,6-dienyl acetate ((*E*)-21)

^{1}H NMR (500 MHz, $CDCl_3$,): δ 5.37-5.33 (m, 2H, 4,8-H), 4.58 (d, J = 7.0 Hz, 2H, 3-H), 3.98 (s, 2H, 10-H), 2.18-2.15 (m, 2H, CH_2), 2.10-2.08 (m, 2H, CH_2), 2.06 (s, 3H, 1-H), 1.71 (s, 3H, 12-H), 1.66 (s, 3H, 11-H) ppm. ^{13}C NMR (125 MHz, $CDCl_3$): δ =171.3 (2-C), 141.8 (5-C), 135.3 (9-C), 125.1 (8-C), 118.7 (4-C), 68.7 (10-C), 61.4 (3-C), 39.1 (6-C), 25.7 (7-C), 21.0 (1-C), 16.4 (12-C), 13.7 (11-C) ppm. IR (ATR): ν = 3298 (b m), 1739 (w), 906 (s), 731 (m), 632 (vs) cm^{-1}. Spectroscopic data were in accordance with ref. [6].

(2*E*,6*E*)-2,6-Dimethylocta-2,6-dien-1,8-diol (8-hydroxygeraniol) (**3**)

Potassium carbonate (78 mg, 0.56 mmol) was added to a solution of 10-hydroxygeranylacetate (*E*)-**21** (100 mg, 0.47 mmol) in methanol (3 mL). After stirring

at room temperature for 2.5 h, the mixture was diluted with water (4 mL) and extracted with diethyl ether (3 x 10 mL). The combined extracts were washed successively with HCl (5 mL, 0.5 M), saturated $NaHCO_3$ (5 mL) and brine (5 mL). The solution was then dried and evaporated under reduced pressure. The crude product was purified by chromatography on silica gel (hexanes / ethyl acetate 1:1) to give (*E*,*E*)-2,6-dimethylocta-2,6-dien-1,8-diol **3** as a colorless oil (70 mg, 0.41 mmol, 88%). ^{1}H NMR (500 MHz, $CDCl_3$,): $\delta =$ 5.39-5.35 (m, 2H, 3,7-H), 4.12 (d, *J* = 6.9 Hz, 2H, 8-H), 3.96 (s, 2H, 1-H), 2.54 (s, 2H, 2 OH), 2.19-2.15 (m, 2H, CH_2), 2.09-2.06 (m, 2H, CH_2), 1.66 (s, 3H, CH_3), 1.65 (s, 3H, CH_3) ppm. ^{13}C NMR (125 MHz, $CDCl_3$): $\delta =$ 138.6 (6-C), 135.1 (2-C), 125.2 (3-C), 123.9 (7-C), 68.6 (1-C), 59.1 (8-C), 39.0 (5-C), 25.6 (4-C), 16.1 (9-C), 13.7 (10-C) ppm. IR (ATR): ν = 3119 (b m), 890 (s), 709 (m), 632 (vs) cm^{-1}. MS (EI): *m*/*z* (%) = 137 (26) [M$^+$ - 33], 123 (10), 121 (16), 116 (18), 109 (12), 95 (24), 86 (26), 84 (97), 78 (24), 75 (16), 69 (34), 68 (100), 67 (68), 65 (20), 55 (28). Spectroscopic data were in accordance with ref. [7].

(*Z*)-3,7-Dimethyl-octa-2,6-dienyl acetate (neryl acetate) ((*Z*)-19)

Acetic anhydride (0.72 mL, 7.70 mmol) was added dropwise to an ice-cooled solution of nerol (1.08 g, 7.00 mmol), dry pyridine (1.47 mL) in dichloromethane (10 mL). After warming to room temperature, the mixture was stirred for 24 h. Water (20 mL) and dichloromethane (20 mL) were then added and the aqueous phase was extracted with dichloromethane (20 mL). The combined organic layers were washed successively with saturated $NaHCO_3$ (10 mL) and brine (10 mL), dried and evaporated under reduced pressure to yield neryl acetate (*Z*)-**19** as a colorless oil (1.20 g, 6.1 mmol, 87%). ^{1}H NMR (250 MHz, $CDCl_3$): $\delta =$ 5.36 (td, *J* = 7.3 and 1.2 Hz, 1H, 8-H), 5.12-5.06 (m, 1H, 4-H), 4.55 (dd, *J* = 7.3 and 0.7 Hz, 2H, 3-H), 2.13-2.06

(m, 6H, 3 CH_2), 2.05 (s, 3H, 1-H), 1.76 (s, 3H, 12-H), 1.68 (s, 3H, 10-H), 1.60 (s, 3H, 11-H) ppm. ^{13}C NMR (125 MHz, $CDCl_3$): $\delta = 171.1$ (2-C), 142.6 (5-C), 132.2 (9-C), 123.6 (8-C), 119.2 (4-C), 61.1 (3-C), 32.2 (6-C), 26.7 (7-C), 25.7 (10-C), 23.5 (12-C), 21.1 (1-C), 17.7 (11-C) ppm. Spectroscopic data were in accordance with ref. [8].

(2*E*,6*Z*)-8-Acetoxy-2,6-dimethylocta-2,6-dienal ((*Z*)-20)

Oxidation of neryl acetate (*Z*)-**19** (396 mg, 2.00 mmol) with selenium dioxide according to the general procedure (t = 5 h) gave after purification by column chromatography (silica gel, eluent: hexanes / ethyl acetate 7 : 3) in a first fraction (R_f = 0.6) (*E*,*Z*)-8-acetoxy-2,6-dimethylocta-2,6-dienal (*Z*)-**20** a colourless oil (58 mg, 0.28 mmol, 14%) and in a second fraction (R_f = 0.3) (*Z*,*E*)-3,7-dimethyl-8-hydroxy-2,6-octa-2,6-dienylacetate (*Z*)-**21** as a colorless oil (175 mg, 0.82 mmol, 41%). ^{1}H NMR (500 MHz, $CDCl_3$): $\delta = 9.40$ (s, 1H, CHO), 6.47 (td, J = 7.6 and 1.3 Hz, 1H, 3-H), 5.44 (t, J = 7.6 Hz, 1H, 7-H), 4.56 (d, J = 7.6 Hz, 2H, 8-H), 2.48 (q, J = 7.6 Hz, 2H, 4-H), 2.32 (t, J = 7.6 Hz, 2H, 5-H), 2.05 (s, 3H, 10-H), 1.80 (s, 3H, CH_3), 1.75 (s, 3H, CH_3) ppm.^{13}C NMR (125 MHz, $CDCl_3$): $\delta = 195.1$ (1-C), 171.0 (9-C), 153.1 (3-C), 140.8 (C_q), 139.8 (C_q), 120.6 (7-C), 60.7 (8-C), 30.5 (5-C), 27.3 (4-C), 23.2 (11-C), 21.0 (10-C), 9.2 (12-C) ppm. Spectroscopic data were in accordance with ref. [9].

(2*Z*,6*E*)-8-Hydroxy-3,7-dimethyl-octa-2,6-dienyl acetate ((*Z*)-21)

^{1}H NMR (300 MHz, $CDCl_3$): $\delta = 5.40$-5.36 (m, 2H, 4,8-H), 4.57 (dd, J = 7.2 and 0.5 Hz, 2H, 3-H), 3.99 (s, 2H, 10-H), 2.17-2.14 (m, 4H, 2 CH_2), 2.05 (s, 3H, 1-H), 1.77 (d, J = 0.9 Hz, 3H, 11-H), 1.67 (s, 3H, 12-H) ppm. ^{13}C NMR (125 MHz; $CDCl_3$): $\delta = 171.2$ (2-C), 141.9 (5-C), 135.6 (9-C), 124.9 (8-C), 119.6 (4-C), 68.8 (10-C), 61.2 (3-C), 31.8

(6-C), 25.9 (7-C), 23.4 (12-C), 21.1 (1-C), 13.7 (11-C) ppm. Spectroscopic data are in accordance with ref. [10].

(2*E*,6*Z*)-2,6-Dimethylocta-2,6-diene-1,8-diol (8-hydroxynerol) (4)

Potassium carbonate (114 mg, 0.88 mmol) was added to a solution of 10-hydroxynerylacetate (*Z*)-**21** (146 mg, 0.69 mmol) in dry methanol (5 mL). After stirring at room temperature for 4 h, the mixture was diluted with water (5 mL) and extracted with diethyl ether (3 x 10 mL). The combined organic layers were washed successively with HCl (5 mL, 0.5 M), saturated $NaHCO_3$ (5 mL) and brine (5 mL). The solution was then dried and evaporated under reduced pressure. The crude product (139 mg) was purified by chromatography on silica gel (hexanes / ethyl acetate 1 : 1) to give (*E*,*Z*)-2,6-dimethylocta-2,6-dien-1,8-diol (8-hydroxynerol) **4** as a colourless oil (86 mg, 0.50 mmol, 73%). ^{1}H NMR (500 MHz, $CDCl_3$): $\delta =$ 5.43-5.39 (m, 2H, 3,7-H), 4.05 (d, *J* = 7.6 Hz, 2H, 8-H), 3.96 (s, 2H, 1-H), 2.83 (s, 2H, 2 OH), 2.18-2.12 (m, 4H, 2 CH_2), 1.75 (s, 3H, CH_3), 1.64 (s, 3H, CH_3); ^{13}C NMR (125 MHz, $CDCl_3$), $\delta =$ 138.2 (6-C), 135.7 (2-C), 125.0 (3-C), 124.8 (7-C), 68.4 (1-C), 58.8 (8-C), 31.4 (5-C), 25.3 (4-C), 23.3 (9-C), 13.8 (10-C) ppm. IR (ATR): ν = 3306 (b m), 1667 (m), 1447 (m), 1377 (m), 997 (s), 887 (s), 710 (m), 632 (vs) cm^{-1}; MS (EI): *m*/*z* (%) = 137 (26) [M^+ - 33], 121 (8), 109 (24), 105 (20), 95 (26), 94 (72), 91 (42), 84 (40), 81 (20), 79 (52), 77 (23), 71 (28), 69 (34), 68 (100), 67 (95), 65 (13), 57 (38), 55 (81), 53 (38). Spectroscopic data were in accordance with ref. [7].

(*cis*)-3,7-Dimethyl-2,3-epoxy-6-octenol (2,3-epoxynerol) (6)

To the suspension of nerol **2** 770 mg, 4.99 mmol) and NaOH solution in water (1.40 g, 140 mL) was added dropwise MPPA (2.28 g, 12.5 mmol) in 50 mL water. The

reaction was stirred at room temp. for 5 h. Then was added brine and the resulting mixture was extracted with ether (3 x 40 mL). The organic layer was washed with brine (2 x 20 mL), dried (Na_2SO_4) and concentrated. The residue was purified by flash chromatography on silica gel (hexanes / diethylether 1 : 1) to give **6** as a colorless oil (656 mg, 3.85 mmol, 77%) and nerol **2** (154 mg, 0.998 mmol, 20%). ^{1}H NMR (500 MHz, DMSO-d_6): δ = 1.23 (s, 3H; 3-CH_3), 1.36-1.42 (m, 1H; 4-H), 1.48-1.53 (m, 1H; 4-H), 1.57, 1.65 (2 x s, 6H; CH_3), 1.99-2.07 (m, 2H; 5-H), 2.77 (dd, *J* = 4.9, 5.9 Hz, 1H; 2-H), 3.39-3.43 (m, 1H; 1-H), 3.53-3.57 (m, 1H; 1-H), 4.87 (t, *J* = 5.6 Hz, 1H; OH), 5.07-5.11 (m, 1H; 6-H) ppm. ^{13}C NMR (125 MHz, DMSO-d_6): δ = 17.3 (9-C), 21.9 (10-C), 23.7 (5-C), 25.4 (8-C), 32.8 (4-C), 59.5 (1-C), 59.9 (3-C), 63.8 (2-C), 123.7 (6-C), 131.0 (7-C) ppm. Spectroscopic data were in accordance with ref. [11].

(2*E*,6*E*)-2,6-Dimethyl-10-oxoundeca-2,6-dienal ((*E*)-22), (*E*)-7-Hydroxy-6,10-dimethyl-undeca-5,9-dien-2-one (11), (5*E*,9*E*)-11-Hydroxy-6,10-dimethyl-undeca-5,9-dien-2-one (13)

Geranylacetone **9** (194 mg, 1 mmol) was oxidised with selenium dioxide according to the general procedure. The crude product was purified by column chromatography (silica gel, eluent: hexanes / ethyl acetate 7 : 3) to give in a first fraction (R_f = 0.7) the starting material **9** as a colourless oil (21 mg, 0.11 mmol, 11%), in a second fraction (R_f = 0.5) (*E*,*E*)-2,6-dimethyl-10-oxoundeca-2,6-dienal (*E*)-**22** as a colourless oil (5 mg, 0.02 mmol, 2%) and in a third fraction (R_f = 0.3) the mixture of (*E*)-7-hydroxy-6,10-dimethyl-undeca-5,9-dien-2-one **11** and (5*E*,9*E*)-11-hydroxy-6,10-dimethyl-undeca-5,9-dien-2-one **13** and as a colourless oil with ratio of (11 : 89, via GC) (72 mg, 0.34 mmol, 34%).

(2*E*,6*E*)-2,6-Dimethyl-10-oxoundeca-2,6-dienal (*E*-22): ^{1}H NMR (300 MHz, $CDCl_3$), $\delta =$ 9.40 (s, 1H, CHO), 6.47-6.48 (m, 1H, CH), 5.15-5.10 (m, 1H, CH), 2.49-2.41 (m, 4H, 2 CH_2), 2.31-2.24 (m, 2H, CH_2), 2.19-2.13 (m, 2H, CH_2), 2.13 (s, 3H, CH_3), 1.74 (d, J = 0.9 Hz, CH_3), 1.65 (s, 3H, CH_3) ppm. Spectroscopic data are in accordance with ref. [12].

(5*E*,9*E*)-11-Hydroxy-6,10-dimethyl-undeca-5,9-dien-2-one (13), (5*E*,9*E*)-7-Hydroxy-6,10-dimethyl-undeca-5,9-dien-2-one (11): ^{1}H NMR (500 MHz, $CDCl_3$), $\delta =$ 5.33-5.31 (m, 1H, CH), 5.10-5.04 (m, 1H, CH), 3.98 (m, 2H, CH_2OH and CHOH), 2.49-2.44 (m, 2H, CH_2), 2.31-2.26 (m, 2H, CH_2), 2.15-1.99 (m, 7H, CH_3 and 2 CH_2), 1.72 (s, 0.3H, CH_3), 1.65 (s, 3H, CH_3), 1.63 (s, 0.3H, CH_3), 1.62 (s, 3H, CH_3) ppm; IR (ATR): ν = 3375 (b m), 1709 (vs), 1439 (m), 1408 (m), 1360 (s), 1243 (m), 1160 (m), 1011 (s), 858 (m), 738 (w), 657 (w), 636 (w) cm^{-1}.

(5*E*,9*E*)-11-Hydroxy-6,10-dimethyl-undeca-5,9-dien-2-one (13): ^{13}C NMR (125 MHz, $CDCl_3$), $\delta =$ 209.3 (2-C), 135.9 (C_q), 134.9 (C_q), 125.5 (CH), 122.9 (CH), 68.8 (4-C), 43.7 (3-C), 39.2 (CH_2), 29.8 (1-C), 25.9 (CH_2), 22.5 (CH_2), 15.9 (CH_3), 13.7 (CH_3) ppm. Spectroscopic data are in accordance with ref. [13].

(5*E*,9*E*)-7-Hydroxy-6,10-dimethyl-undeca-5,9-dien-2-one (11): ^{13}C NMR (125 MHz, $CDCl_3$): $\delta =$ 208.6 (2-C), 138.1 (C_q), 134.6 (C_q), 124.1 (CH), 120.1 (CH), 76.9 (7-C), 43.3 (3-C), 34.2 (CH_2), 29.9 (1-C), 25.9 (CH_2), 22.0 (CH_2), 18.0 (CH_3), 11.7 (CH_3) ppm. Spectroscopic data are in accordance with ref. [14].

(2*E*,6*Z*)-2,6-Dimethyl-10-oxo-undeca-2,6-dienal ((*Z*)-22), (*Z*)-7-Hydroxy-6,10-dimethyl-undeca-5,9-dien-2-one (12), (5*Z*,9*E*)-11-Hydroxy-6,10-dimethyl-undeca-5,9-dien-2-one (14)

Nerylacetone **10** (194 mg, 1 mmol) was oxidised with selenium dioxide according to the general procedure. The crude product was purified by column chromatography

(silica gel, eluent: hexanes / ethyl acetate 7 : 3) to give in a first fraction (R_f = 0.7) the starting material **10** as a colourless oil (8 mg, 0.04 mmol, 4%), in a second fraction (R_f = 0.5) (2*E*,6*Z*)-2,6-dimethyl-10-oxoundeca-2,6-dienal (*Z*)-**22** as a colourless oil (10 mg, 0.05 mmol, 5%) and in a third fraction (R_f = 0.3) the mixture of (*Z*)-7-hydroxy-6,10-dimethyl-undeca-5,9-dien-2-one **12** and (5*Z*,9*E*)-11-hydroxy-6,10-dimethyl-undeca-5,9-dien-2-one **14** as a colourless oil with a ratio of (14 : 86, via GC) (90 mg, 0.43 mmol, 43%).

(2*E*,6*Z*)-2,6-Dimethyl -10-oxoundeca-2,6-dienal (Z-22): ^{1}H NMR (300 MHz, $CDCl_3$): δ = 9.40 (s, 1H, CHO), 6.51-6.45 (m, 1H, 3-H), 5.16-5.12 (m, 1H, 7-H), 2.49-2.41 (m, 4H, 2 CH_2), 2.30-2.22 (m, 4H, 2 CH_2), 2.13 (s, 3H, 11-H), 1.75 (d, *J* = 0.9 Hz, 3H, 13-H), 1.71-1.68 (m, 3H, 12-H) ppm; ^{13}C NMR (125 MHz, $CDCl_3$), δ = 208.3 (10-C), 195.2 (1-C), 153.2 (3-C), 139.6 (C_q), 134.8 (C_q), 124.8 (7-C), 43.6 (9-C), 30.2 (CH_2), 30.1 (11-C), 27.3 (CH_2), 23.1 (12-C), 22.2 (CH_2), 9.2 (13-C) ppm; IR (ATR): ν = 3287 (w), 2973 (w), 2878 (w), 2353 (w), 2252 (w), 1739 (w), 906 (vs), 731 (m), 632 (vs) cm^{-1}.

(5*Z*,9*E*)-11-Hydroxy-6,10-dimethyl-undeca-5,9-dien-2-one (14), (*Z*)-7-Hydroxy-6,10-dimethyl-undeca-5,9-dien-2-one (12): ^{1}H NMR (500 MHz, $CDCl_3$), δ = 5.41-5.38 (m, 1H, CH), 5.37-5.31 (m, 0.2H, CH*), 5.09-5.07 (m, 1.2H, CH and CH*), 3.98 (s, 2H, CH_2OH), 3.96-3.95 (m, 0.2H, CHOH*), 2.51-2.48 (m, 0.4H, CH_2*), 2.32-2.21 (m, 2.4H, CH_2 and CH_2*), 2.17-2.06 (m, 6H, CH_3, CH_3*, 2 CH_2 and CH_2*), 1.71 (s, 0.6H, CH_3*), 1.68 (s, 3H, CH_3), 1.63 (s, 0.6H, CH_3), 1.60 (s, 0.6H, CH_3*) ppm (the protons* are attributed to compound **12**); IR (ATR): ν = 3316 (b w), 1717 (w), 892 (s), 717 (m), 632 (vs) cm^{-1}.

(5*Z*,9*E*)-11-Hydroxy-6,10-dimethyl-undeca-5,9-dien-2-one (14): ^{13}C NMR (125 MHz, $CDCl_3$), δ = 209.2 (2-C), 136.1 (C_q), 135.1 (C_q), 125.2 (CH), 123.7 (CH), 68.6

(4-C), 43.9 (3-C), 31.5 (CH_2), 30.0 (1-C), 25.7 (CH_2), 23.2 (CH_3), 22.4 (CH_2), 13.6 (CH_3) ppm. Spectroscopic data are in accordance with ref. {Seifert, 2009 #24}.

(*Z*)-7-Hydroxy-6,10-dimethyl-undeca-5,9-dien-2-one (12): ^{13}C NMR (125 MHz, $CDCl_3$), $\delta = 208.7$ (2-C), 138.1 (C_q), 134.6 (C_q), 124.1 (CH), 120.1 (CH), 76.9 (7-C), 43.3 (3-C), 34.2 (CH_2), 29.7 (1-C), 25.9 (CH_2), 22.0 (CH_2), 18.0 (CH_3), 11.7 (CH_3) ppm.

References

1. Bühler, C.; Le-Huu, P.; Urlacher, V. B. *ChemBioChem.* **2013,** *14,* 2189-2198.
2. Girhard, M.; Tieves, F.; Weber, E.; Smit, M. S.; Urlacher, V. B. *Appl. Microbiol. Biotechnol.* **2013,** *97,* 1625-1635.
3. Watanabe, Y.; Laschat, S.; Budde, M.; Affolter, O.; Shimada, Y.; Urlacher, V. B. *Tetrahedron* **2007,** *63,* 9413-9422.
4. Seifert, A.; Vomund, S.; Grohmann, K.; Kriening, S.; Urlacher, V. B.; Laschat, S.; Pleiss, J. *ChemBioChem.* **2009,** *10,* 853-861.
5. Suhara, Y.; Hirota, Y.; Nakagawa, K.; Kamao, M.; Tsugawa, N.; Okano, T. *Bioorg. Med. Chem.* **2008,** *16,* 3108-3117.
6. Meinwald, J.; Thompson, W. R.; Eisner, T.; Owen, D. F. *Tetrahedron Lett.* **1971,** *12,* 3485-3488.
7. Fretz, H.; Woggon, W.-D. *Helv. Chim. Acta.* **1986,** *69,* 1959-1970.
8. Chiarotto, I.; Feroci, M.; Sotgiu, G.; Inesi, A. *Eur. J. Org. Chem.* **2013,** *2013,* 326-331.
9. Lavoie, R.; Toró, A.; Deslongchamps, P. *Tetrahedron* **1999,** *55,* 13037-13050.
10. Marshall, J. A.; Lebreton, J. *J. Org. Chem.* **1988,** *53,* 4108-4112.

11. Fringuelli, F.; Germani, R.; Pizzo, F.; Santinelli, F.; Savelli, G. *J. Org .Chem.* **1992,** *57,* 1198-1202.
12. Labadie, G. R.; Viswanathan, R.; Poulter, C. D. *J. Org. Chem.* **2007,** *72,* 9291-9297.
13. McMurry, J. E.; Matz, J. R.; Kees, K. L. *Tetrahedron* **1987,** *43,* 5489-5498.
14. Müller, A.; Abraham, W. R.; Kieslich, K. *Bull. Soc. Chim. Belg.* **1994,** *103,* 405-423.
